# Organism body size structures the soil microbial and nematode community assembly at a continental and global scale

**DOI:** 10.1038/s41467-020-20271-4

**Published:** 2020-12-17

**Authors:** Lu Luan, Yuji Jiang, Menghua Cheng, Francisco Dini-Andreote, Yueyu Sui, Qinsong Xu, Stefan Geisen, Bo Sun

**Affiliations:** 1 State Key Laboratory of Soil and Sustainable Agriculture, Institute of Soil Science, Chinese Academy of Sciences, 210008 Nanjing, China; 2 University of Chinese Academy of Sciences, 100049 Beijing, China; 3 College of Life Science, Nanjing Normal University, 210023 Nanjing, China; 4 Department of Plant Science & Huck Institutes of the Life Sciences, The Pennsylvania State University, University Park, PA USA; 5 Northeast Institute of Geography and Agricultural Ecology, Chinese Academy of Sciences, 150040 Harbin, China; 6 Laboratory of Nematology, Wageningen University, 6700 ES Wageningen, Netherlands

**Keywords:** Microbial ecology, Theoretical ecology

## Abstract

Body size is a key life-history trait that influences community assembly by affecting how ecological processes operate at the organism level. However, the extent to which the relative influences of ecological processes mediate the assembly of differentially sized soil organisms is still unknown. Here, we investigate the community assembly of differentially sized soil microorganisms and microfauna using a continental-scale sampling effort combined with a global-scale meta-analysis. Our results reveal a general relationship between organism body size and the stochastic-deterministic balance operating on community assembly. The smallest microorganisms (bacteria) are relatively more influenced by dispersal-based stochastic processes, while larger ones (fungi, protists and nematodes) are more structured by selection-based deterministic processes. This study elucidates a significant and consistent relationship between an organism life-history trait and how distinct ecological processes operate in mediating their respective community assemblages, thus providing a better understanding of the mechanisms supporting soil biodiversity.

## Abstract (web summary)

It is unclear whether body size affects community assembly mechanisms of soil biota. Here, the authors analyse soil microbial and nematode communities sampled along a 4000-km transect in China and global soil microbiome data to show that bacterial assembly is governed by high dispersal, whereas larger taxa are more influenced by deterministic processes.

## Introduction

Soil biodiversity is extremely complex and diverse, with millions of species and billions of individual organisms being found within a handful of soil; this includes microscopic bacteria, fungi and protists, as well as soil animals including nematodes, microarthropods, and earthworms^1^. These organisms and the composition of this biodiversity are underlying major ecosystem processes such as carbon and nutrient flow^2^. As such, understanding the formation and maintenance mechanism of soil biodiversity, namely community assembly, is central to predicting the taxonomic and functional variation in soils^2–4^. We have rapidly increased our understanding of spatial and environmental factors structuring the distribution of many soil organism groups, such as bacteria, fungi, protists, and nematodes^5–8^. However, we still lack a comprehensive understanding of how life-history traits, such as body size, relate to community assembly processes among distinct groups of soil (micro)organisms^9–11^.

**Fig. 1:**
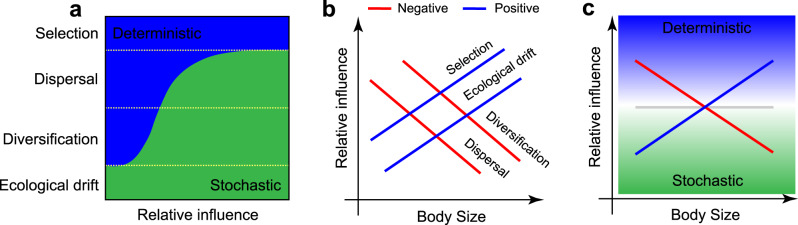
The conceptual framework for the study. Conceptual model displaying (**a**) the extent at which the four classes of ecological processes (selection, dispersal, ecological drift, and diversification; sensu Vellend^12^) relates to the stochastic-deterministic balance. Here, selection is an exclusively deterministic process, whereas drift is exclusively stochastic. Dispersal and diversification are partially stochastic and partially deterministic. This occurs because both dispersal and diversification rates can be influenced by deterministic organism’s life-history traits (e.g., spore-forming organisms and horizontal gene transfer, respectively), as well as by random factors (e.g., passive dispersal and mutation); **b** a hypothetical visualization of the relative influence of each process according to distinct organism body sizes. Selection and ecological drift are more prone to influence organisms with bigger body sizes due to their relatively lower population sizes. On the other hand, diversification and dispersal is conceptualized to exert a higher influence on organisms with smaller body sizes. Despite selection and ecological drift opposition in the stochastic-deterministic spectrum (plot a), we hypothesized that **c** the effect of selection might override that of ecological drift in organisms with bigger body sizes, thus resulting in a greater deterministic signal. Conversely, we conceptualize that random dispersal can have an overriding effect on the assembly of organisms with smaller body sizes, thus resulting in a greater stochastic signal.

Deterministic and stochastic processes have become broadly recognized as two mutually non-exclusive determinants of community assembly that simultaneously operate on the maintenance of species diversity at the local and global scale^12–18^. Deterministic processes structure community assemblages by operating on fitness differences across taxa (e.g., survival, growth, and reproduction), while stochastic processes are mostly characterized by random changes in community structures with respect to species identities and/or functional traits^13–15^. A conceptual synthesis in community ecology proposes that any given ecological community is dynamically structured by an interplay of four ecological processes (selection, dispersal, ecological drift, and diversification)^12^, all of which fall within the deterministic-stochastic framework (Fig. 1a). Selection (often termed as habitat filtering) is a deterministic process that operates by filtering out non-adapted species within a given community^9,13,14^. Ecological drift is a stochastic process that eliminates species via random birth, death, and reproduction events^9,14^. Dispersal is the movement of organisms across space, which can be either deterministic (via predictable differences in dispersal ability between species due to specific life-history traits) or stochastic (passive dispersal by wind, water, or hitchhiking on animals)^9,14^. Diversification, i.e., the generation of new genetic variation, is widely accepted as a stochastic process, however, this process can also be considered deterministic when mediated by specific organism traits that enhance or diminish evolutionary rates between species^14^.

Specific organism traits directly relate to how distinct ecological processes operate on community assembly. Among those traits, body size is arguably one of the most important^19–21^. Community assembly processes have been shown to differ between communities of species with different body sizes (Fig. 1b, c). Within the soil microbiome, bacterial cells are the smallest ones. These organisms are often associated with the degradation of diverse and complex organic compounds, and display relatively high environmental tolerance compared with other microorganisms^22–25^. Smaller organisms generally have fast growth rates that potentially increases mutation and evolutionary rates^14,26^. The higher population sizes and lower sinking rates of organisms with small body sizes are known to increase their potential for passive dispersal^20–22,27^. Also, such characteristics reduce extinction risks and the influence of ecological drift^20,28^. In this study, we hypothesized that the relative influence of distinct ecological processes (here evaluated as the deterministic-stochastic balance)^13^ vary in mediating the assembly of soil communities according to distinct organism body sizes. Specifically, we expect a lower influence of dispersal and a higher influence of selection to mediate the community assembly of organisms with larger body sizes. Similarly, we expect the community assembly of organisms with smaller body sizes to be greatly influenced by stochastic processes.

Here, we seek to investigate the extent to which distinct ecological processes mediate the community assembly of differentially sized soil organisms including bacteria, fungi, protists, and nematodes. We use a continental scale environmental sampling approach combine with a global-scale meta-analysis to answer the following questions: (i) is there a significant relationship between organism body size and distance-decay patterns of microbial groups in paddy soils? (ii) what are the relative influences of stochastic and deterministic processes mediating the community assembly of soil organism groups with different body sizes? and (iii) is there a global and consistent relationship between organism body size and the stochastic-deterministic balance across ecosystems? To address the first two questions, we sample paddy soils along a north-south transect at a continental scale (spanning 4165 km), which represents the third largest cropland area and the largest anthropogenic wetland on Earth^29,30^, and compare the community assembly processes of multiple differentially sized microbial groups (28 groups). As an effective and reliable method to quantify rates of diversification and ecological drift is still lacking, we focus attention mostly on the importance of environmental selection and dispersal. Last, our meta-analysis provides supporting evidences and robustness to our findings by indicating that the general patterns observed in our transect dataset are consistent across ecosystems at a global scale. For example, flooded soil environments may promote the growth and dispersal of most protists but suppress the growth of several fungal groups. In contrast, such effects on growth promotion and suppression may be absent (or smaller) in drylands^31,32^. Therefore, the meta-analysis is key to support that – albeit intrinsic ecosystem dynamics might differ – the relationship between body size and the balance of community assembly processes across multiple ecosystems is consistent rather than idiosyncratic. Our findings consistently support a tight correlation between organism body size and the stochastic-deterministic balance. Smaller organisms displayed high dispersal rates leading to a major impact of stochastic processes in predicting their communities, while larger organisms showed lower dispersal rates leading to an increased importance of environmental selections in determining their communities.

## Results

### Community structure and body size distribution of soil microbiomes

We obtained 26,994 bacterial, 29,312 fungal, and 16,089 protist sequences per sample after excluding singletons and rarefying to even depth. These sequences were clustered into 10,379 bacterial, 3541 fungal, and 4716 protistan OTUs, respectively. Higher-level taxonomic assignment of the sequences indicated the presence of 47 bacterial groups, 27 fungal groups, and 39 protistan groups (Supplementary Fig. 1). Twenty-eight groups with relative abundance ≥1% and presence ≥80% of all samples were selected for downstream analyses. This resulted in 10 bacterial groups accounting for 95.5% of all bacterial sequences (average relative abundance was between 2.6% (Gemmatimonadetes) and 27.1% (Actinobacteria)), 7 fungal groups accounting for 79.8% of all fungal sequences (average relative abundance was between 1.1% (Chytridiomycota) and 21.1% (Eurotiomycetes)), and 11 protistan groups accounting for 80.8% of all protistan sequences (average relative abundance was between 1.3% (Discosea) and 22.9% (Glissomonadida)).

**Fig. 2:**
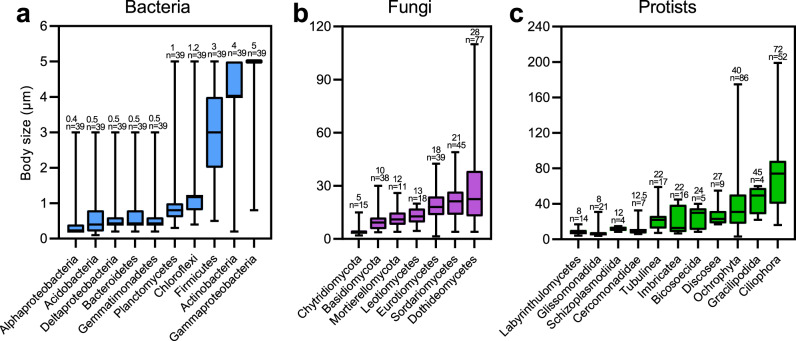
Taxa and body sizes of soil microorganisms. The average body size of 28 organism groups, including bacteria (10 groups), fungi (7 groups), and protists (11groups). Box plots indicate median (middle line), 25th, 75th percentile (box) and 5th and 95th percentile (whiskers). *n* represents the number of samples.

To determine the body size of each organism group, literature documenting body sizes of the assigned species were collected (Fig. 2 and Supplementary Data 1). Our results showed that body sizes of the 28 selected groups in paddy soils ranged from 0.4 μm to 72 μm, bacterial cells vary between 0.4 μm (Alphaproteobacteria) and 5 μm (Gammaproteobacteria), fungal spores between 5 μm (Chytridiomycetes) and 28 μm (Dothideomycetes), and protistan cells between 8 μm (Labyrinthulomycetes) and 72 μm (Ciliophora). Chao1 index and richness were used to estimate alpha diversity of the 28 selected dominant groups (Supplementary Fig. 2), with the bacterial Chloroflexi having highest diversity and Gemmatimonadetes having lowest. For fungal communities, Sordariomycetes contained the highest diversity and Mortierellomycota the lowest. For protistan communities, Glissomonadida was most diverse and Labyrinthulomycetes was least diverse. We observed that the Chao1 index and richness were significantly negatively correlated with the logarithms of body sizes (*R*^*2*^ = 0.30, *P* < 0.05 and *R*^*2*^ = 0.28, *P* < 0.05; Supplementary Fig. 3).

### Distance-decay relationship and variation partitioning

**Fig. 3:**
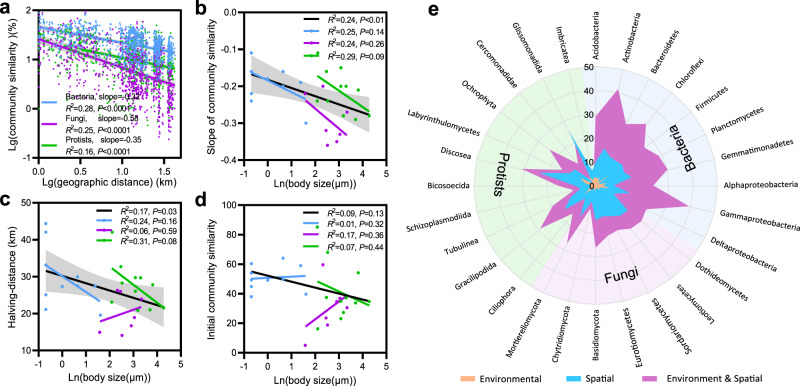
Distance-decay relationship and variation partitioning analysis of soil microbiomes across distinct organism body sizes. **a** Distance-decay relationship regressions based on log-transformed Bray-Curtis similarities between pairs of communities and log-transformed geographic distances between sampling sites. Linear correlations between the slopes (**b**), halving-distance (**c**), and initial community similarity (**d**) of the distance-decay relationships and the logarithm of body sizes for the 28 organism groups, including 10 bacterial groups, 7 fungal groups, and 11 protistan groups. The statistics are for the regression of all data points. Lines represent the least squares regression fits and shaded areas represent the 95% confidence intervals. We applied one-sided F test and two-sided *t* test, and the calculated *P* values as shown. **e** Variation in composition of each organism group partitioned into pure environmental factors, spatial factors, and combined factors.

Distance-decay relationships (DDRs) were used to estimate the decline in community similarity of bacteria, fungi, and protists across eastern Asia spanning a geographic distance of 4165 km (Fig. 3a). DDRs for all three organism groups were significant (*P* < 0.001), with slope being steeper for fungi (slope = −0.58, *R*^*2*^ = 0.25) than for bacteria (slope = −0.32, *R*^*2*^ = 0.28) and protists (slope = −0.35, *R*^*2*^ = 0.16). We further observed consistently significant DDRs for all 28 selected groups (Supplementary Fig. 4). Slopes of DDRs for bacteria were between −0.11 (Alphaproteobacteria) and −0.30 (Gammaproteobacteria), for fungi between −0.20 (Basidiomycota) and −0.36 (Leotiomycetes), and for protists between −0.11 (Labyrinthulomycetes) and −0.29 (Ochrophyta). Across 28 organism groups, we observed that both the distance-decay slope (*R*^*2*^ = 0.24, *P* < 0.01) and the halving-distance (*R*^*2*^ = 0.17, *P* < 0.05) were significantly negatively correlated with the logarithms of body size, but the initial similarity did not significantly correlate with body size (*R*^*2*^ = 0.09, *P* = 0.13; Fig. 3b–d). The relative influence of environmental and spatial gradients on bacterial, fungal, and protistan communities was measured by variation partitioning analysis (Fig. 3e and Supplementary Table 1). Pure environmental factors including MAT, MAP, and pH explained 2.6%, 1.7%, and 1.1%, while spatial factors explained 9.8%, 12.8%, and 8.9% of the variance in bacterial, fungal, and protistan communities, respectively. For the 28 selected organism groups, spatial factors explained more of the variation (2.6–19.5%) than environmental factors (1.2–7.3%). For half of the selected 28 groups, only spatial factors significantly explained their variance (5.7–15.0%). Generally, variations in communities were largely explained by combined spatial and environmental factors (2.9–25.7%), implying a high auto-correlation between spatial factors and environment factors such as MAT, MAP, and pH (*P* < 0.05, Supplementary Fig. 5).

### Community assembly mechanisms of soil microorganisms in paddy soils

**Fig. 4:**
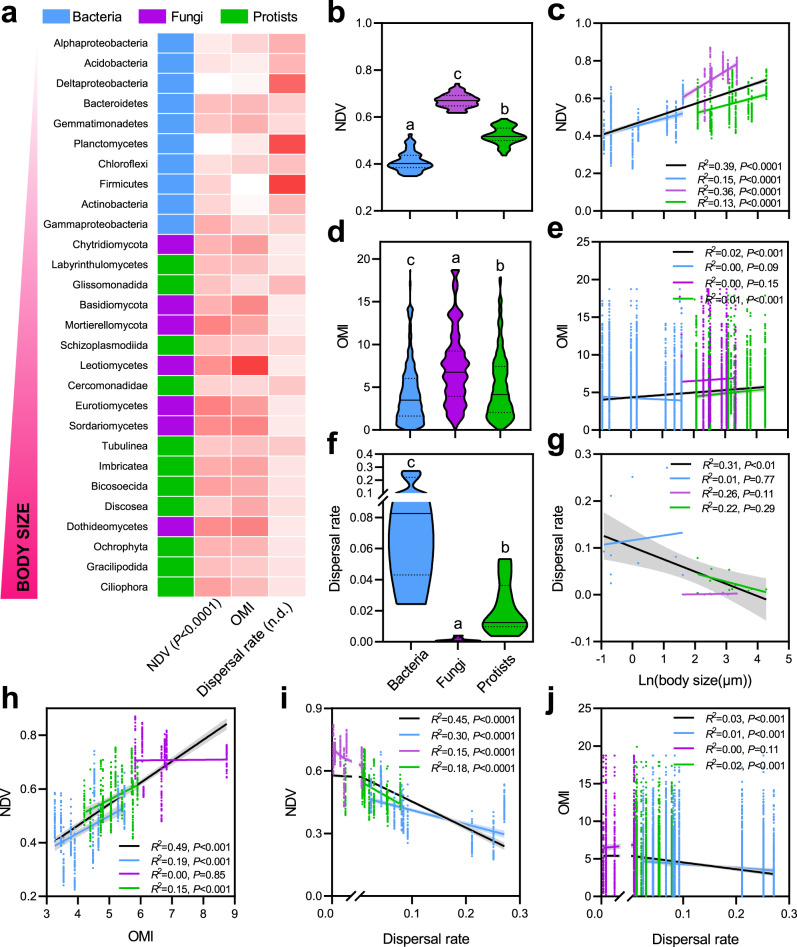
Community assembly processes of soil organisms in paddy systems. **a** Heat map displaying the null deviation value (NDV), outlying mean index (OMI), and dispersal rate of the 28 organism groups. Darker colors indicate bigger values, and n.d. indicates absence of correlation. Violin plots displaying NDV (**b**), OMI (**d**), and dispersal rate (**f**) of bacteria, fungi, and protists. The lowercase indicates significant difference between groups (*P* < 0.05) via one-way ANOVA and Tukey’s post-hoc tests. Correlations between the logarithm of body sizes and NDVs (**c**), OMIs (**e**), and dispersal rates (**g**); correlations between NDVs and OMIs (**h**) and dispersal rates (**i**); and correlations between OMIs and dispersal rates (**j**). Significant correlations were found using all 28 organism groups, bacterial groups, fungal groups, and protistan groups. The statistics are for the regression of all data points. Lines represent the least squares regression fits and shaded areas represent the 95% confidence intervals. We applied one-sided *F* test and two-sided *t* test, and the calculated *P* values as shown.

The abundance-based β-null model was used to quantify the relative influences of distinct community assembly processes^33^. High and low null deviation values (NDVs) indicate greater influence of deterministic and stochastic processes, respectively. The results showed that all three organism groups deviated from neutral predictions, with fungal (avg.NDVs = 0.67) and protistan (avg.NDVs = 0.52) communities displaying significantly higher beta-null deviations than communities of bacteria (avg.NDVs = 0.41, *P* < 0.001; Fig. 4a, b). The NDVs of soil organisms were significantly positively correlated with their logarithms of body sizes (*R*^*2*^ = 0.39, *P* < 0.001, Fig. 4c). This positive correlation also existed within bacterial (*R*^*2*^ = 0.15, *P* < 0.001), fungal (*R*^*2*^ = 0.36, *P* < 0.001), and protistan groups (*R*^*2*^ = 0.13, *P* < 0.001).

The niche breadth was calculated to evaluate the influence of selection on community assembly. Low outlying mean index (OMI) value indicates wide niche breadth, and high OMI value indicates narrow niche breadth^34^. The average OMI of fungal communities (7.37) was higher than that of bacterial (4.28) and protistan (5.08) communities (Fig. 4a, d, *P* < 0.05). Body sizes of soil organism groups were significantly positively correlated with the corresponding OMIs (*R*^*2*^ = 0.02, *P* < 0.001), albeit relationships were weaker within taxonomic groups (Fig. 4e). The OMIs were significantly positively correlated with NDVs (*R*^*2*^ = 0.49, *P* < 0.001; Fig. 4h).

We calculated the dispersal rate (*m*) for each OTU by fitting the frequency distribution of the data to the Sloan neutral model^35^. Across the 28 selected organism groups, the bacterial groups (avg.*R*^*2*^ = 0.49) could always be better fitted to the neutral model than fungal (avg.*R*^*2*^ = 0.09) and protistan (avg.*R*^*2*^ = 0.22) groups (Supplementary Table 2). The bacterial community presented significantly higher dispersal rates (*m* = 0.10) than fungal (*m* = 0.01) and protistan (*m* = 0.02) communities (*P* < 0.05; Fig. 4 a, f and Supplementary Table 2). We detected a significantly negative correlation between the logarithms of body sizes and dispersal rates (*R*^*2*^ = 0.31, *P* < 0.001) across the 28 selected organism groups; however, no significant correlation was found within taxonomic groups (*R*^*2*^ = 0.01−0.26, *P* > 0.05; Fig. 4g). Furthermore, dispersal rates were significantly negatively correlated with NDVs (*R*^*2*^ = 0.45, *P* < 0.001; Fig. 4i) and there was a significant positive correlation between dispersal rates and OMIs (*R*^*2*^ = 0.03, *P* < 0.001; Fig. 4j).

### Meta-analysis: community assembly mechanisms of soil organisms at global scale

**Fig. 5:**
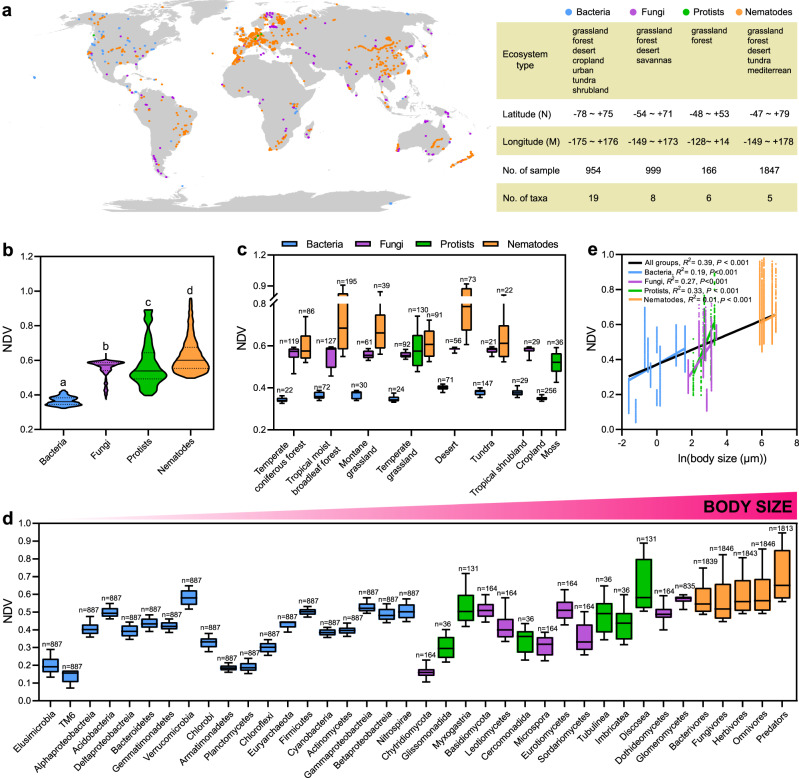
Community assembly processes of soil organisms at the global scale. **a** Sampling locations from where data were obtained for bacteria, fungi, protists, and nematodes. Null deviation values (NDVs) of different organism groups in the global dataset (**b**) and separated by ecosystem types (**c**), respectively. **d** NDVs of organisms (38) with varying body sizes. The lowercase indicates significant difference between groups (*P* < 0.05) via one-way ANOVA and Tukey’s post-hoc tests. Box plots indicate median (middle line), 25th, 75th percentile (box), and 5th and 95th percentile (whiskers). *n* represents the number of samples. **e** Global relationships between NDVs and organism body sizes across and within different organism groups. The statistics are for the regression of all data points. Lines represent the least squares regression fits and shaded areas represent the 95% confidence intervals. We applied one-sided *F* test and two-sided *t* test, and the calculated *P* values as shown. The global dataset of bacterial communities (954 datasets) was obtained from Thompson et al.^5^. The fungal dataset (999 datasets) was obtained from Bahram et al.^2^ and Davison et al.^65^. For protists, the dataset of the protistan community (166 datasets) was obtained from Fiore-Donno et al.^66^ and Heger et al.^67^. The dataset of nematode communities (1847 datasets) was obtained from van den Hoogen et al.^8^.

To provide a deeper general insight into the community assembly processes across different body sizes, we compared the NDVs of organisms with varying body sizes using previous published global data (ranging from −78 N to +79 N, including ~3966 data sets containing bacteria, fungi, and protists as well as nematodes; Fig. 5a). Our datasets covered multiple ecosystems, such as grasslands, forests, tundra, deserts, and shrublands (Fig. 5b, c). Generally, the average NDV value of nematodes (0.63) was significantly greater than that of protists (0.59), fungi (0.55), and bacteria (0.37) (*P* < 0.001). Specifically, for almost all organism groups, the highest NDV was found in deserts (0.40, 0.34, and 0.77 for bacteria, fungi, and nematodes, respectively) and temperate grasslands for protists (0.59); the lowest NDV for bacteria, fungi, protists, and nematodes occurred in temperate coniferous forests (0.34), temperate moist broadleaf forests and temperate coniferous forests (0.55), bog woodland (0.30), and temperate moist broadleaf forests (0.60), respectively.

The body size of the 38 selected groups in metadata ranged from 0.2 to 904 μm (Fig. 5d and Supplementary Data 1). The average cell/body lengths of bacteria (19 groups) ranged from 0.2 μm (Elusimicrobia) to 5 μm (Nitrospirae), of fungi (8 groups) from 5 μm (Chytridiomycota) to 130 μm (Glomeromycetes), of protists (6 groups) from 8 μm (Glissomonadida) to 27 μm (Discosea), and of nematodes (5 groups) from 357 μm (Bacterivores) to 904 μm (Predators). The greatest NDV occurred in bacterial Verrucomicrobia (0.58), fungal Glomeromyceta (0.57), protistan Discosea (0.64), and nematode predators (0.71); the lowest NDV occurred in bacterial TM6 (0.14), fungal Chytridiomycota (0.16), protistan Myxogastria (0.54), and nematode bacterivores (0.58; Fig. 5d and Supplementary Fig. 6).

A significant positive correlation occurred between NDV and the logarithms of body sizes across all groups (*R*^*2*^ = 0.39, *P* < 0.001), which also existed within bacterial (*R*^*2*^ = 0.19, *P* < 0.001), fungal (*R*^*2*^ = 0.27, *P* < 0.001), protistan (*R*^*2*^ = 0.33, *P* < 0.001), and nematode groups (*R*^*2*^ = 0.01, *P* < 0.001; Fig. 5e). These relationships also appeared in all ecosystems for all selected groups (*P* < 0.001; Supplementary Fig. 7), with slopes ranging from 0.043 (boreal forests) to 0.075 (Mediterrean), and with the *R*^2^ ranging from 0.22 (tundra) to 0.49 (Mediterrean). For bacterial, fungal, and protistan groups, positive relationships between body sizes and NDVs were found in all observed ecosystems (*P* < 0.001), with *R*^*2*^ ranging from 0.16 (desert) to 0.31 (temperate conifer forests) for bacterial communities, 0.14 (savannas) to 0.29 (boreal forests) for fungal communities, and 0.15 (temperate grasslands) to 0.69 (blanket bog) for protistan communities. For nematodes, significant relationships were observed in most ecosystems except in tundra with *R*^2^ ranging from 0.03 (tropical moist forests) to 0.16 (temperate grasslands).

## Discussion

We used a continental soil survey and a global meta-analysis to unravel a consistent pattern, where the relative importance of community assembly processes varies depending on body sizes across distinct soil organism groups. In particular, we found that organism body sizes underlie community assembly processes. The results confirm our hypothesis that smaller microorganisms are greatly influenced by stochasticity, whereas communities of larger organisms are mostly structured by deterministic processes. This indicates that selection might play a major role in structuring the communities of larger organisms, for instance, via changes in dispersal limitation and/or by imposing distinct levels of (a)biotic constraints.

Distance-decay relationships (DDRs) were present for all tested microbial groups, indicating that microorganisms are not ubiquitously distributed across space. The biogeographical patterns of the different groups of soil microorganisms differed significantly in the extent to which they are influenced by environmental selection, as opposed to dispersal limitation. The DDR slope steepened and halving-distance shortened with enlarged body size (Fig. 3), highlighting that body size determined the observed biogeographical patterns. Variation partitioning analysis revealed that spatial factors rather than environmental ones were consistently important drivers of the observed variability in microbial communities (Fig. 3e). Contrary to previous studies^10,11,36^, our results point towards the prominence of dispersal limitation structuring the microbiome assembly in paddy soils. It is likely that such discrepancy across studies relates to the scale at which communities are investigated, the so-called ‘scale-dependency’^13,37,38^. As pointed out in these studies, it is important realizing that modeling how distinct assembly processes operate in structuring community types is fundamentally dependent on the scale at which communities are investigated. Besides, we can also argue that similar management regimes used in paddy fields, such as constant flooding, long-term regular dry-wet cycles, and the same crop cultivation (rice), may have consistently created relatively homogeneous microhabitats that overall reduce environmental differences across study sites^30,39^. However, we cannot rule out the possible importance of unmeasured variables (including abiotic and biotic ones) accounting for the observed differences in the microbial communities^17,23^.

The β-null model showed a significant relationship between body size and community assembly processes, with smaller microorganisms being more strongly influenced by stochasticity (Fig. 4). We believe this should be closely related to the discrepant dispersal potentials of different body-sized microorganisms. Indeed, we found a gradual decrease in dispersal rate with increased body size (0.4−72 μm; Fig. 4), confirming the fact that body size affects dispersal rates^40^. Although most studies consider dispersal as a stochastic process^41^, we categorized dispersal as being both deterministic and stochastic, because dispersal rates rely primarily on body size as an intrinsic trait that influences both passive and active dispersal. Some discrepancies were found in dispersal rates for distinct microbial groups; for example, some large-sized protists showed higher dispersal rates than smaller fungi. The methodological biases might have contributed to this finding including a potential underestimation of fungal sizes which can form large-sized hyphae that potentially limit dispersal potential^21,31^.

The gradual decrease in stochasticity with the increase in organism body size (Figs. 4 and 5) is largely a consequence of environmental selection. This is supported by the significant relationships between body sizes and with the corresponding OMIs (Fig. 4). However, environmental selection only plays a minor role in controlling the microbiome assembly in paddy fields (Fig. 3). In this system, dispersal plays a major role by offsetting the variation in community imposed by selection or drift. It can be argued that in paddy soils, dispersal largely determines the direction of microbiome community assembly^20,22,23^. Interestingly, we noticed that protists with larger body sizes are more influenced by stochasticity than fungi. This body-size discrepancy might be attributed to a limited importance of fungi in the continuously flooded paddy systems^31^, whereas protists that are mainly determined by soil water content thrive in these systems^7,32^. In fact, protistan communities in our study were dominated by many typical aquatic taxa. Alternatively, hyphal dynamics of fungi are more prone to be determined by selection, for instance, due to competition^31^. On the other hand, spore structures represent only a short fraction of the fungal life cycle. Fungal spores are mostly associated with dispersal and/or environmental persistence under unfavorable environmental conditions.

The meta-analysis including all three microbial groups and nematodes further confirmed that the significant relationship between body size and community assembly occurred not only across organism groups, but also within the four groups at a global scale and within diverse ecosystems (Fig. 5). We noticed that this relationship differed in height and slope in diverse ecosystems, which might be related to the discrepant balance between community assembly processes in various ecosystems. Comparatively to other ecosystems, a higher influence of deterministic processes in desert soils is likely due to strong environmental selection imposed by harsh climate conditions (high temperature and low moisture), and limited nutrient availability. On the other hand, a higher influence of stochastic processes in temperate coniferous forests emerges likely as a consequence of lower stringency of environmental selection and higher habitat patchiness as a result of biodiversity and system complexity^8,42,43^. Consistent with our results, the meta-analysis revealed a relative high proportion of stochasticity in agroecosystems, likely due to the regular management of the system that allow for a dynamic community turnover and lowered homogeneity of the system over time^44^.

Notably, the deviation in determination of body size may bring some biases to the accuracy of results and would potentially mask the general relationship between community assembly and body sizes. For example, most fungi included here have different life forms such as hyphal-forming, yeasts and spores^21^. Therefore, the main challenge for microbial empiricists now is to create reliable high-resolution and high-throughput body-size detection and corresponding taxonomic-identification (morphological and molecular methods) systems to specifically test how body size predicts community assembly. Additionally, body sizes vary greatly within each taxonomic group, but this pattern should be mitigated by much greater between-group than within-group variation^37,45,46^. As such, our finding of a linear relationship between the community assembly and body size should be robust. It is worth emphasizing that the findings in this study support a general relationship between body size and how distinct ecological processes mediate community assembly. However, it is important noting that caution is warranted in inferring processes from patterns in ecology. Further analytical developments and experimental studies are needed to corroborate our findings and fine-partition this relationship across other gradients and ecosystem types.

We identified a widespread mechanism between body size and community assembly both across and within organism groups of the soil organisms (including bacteria, fungi, protists, and nematodes). In particular, our results revealed that communities of smaller microorganisms (i.e., bacteria) are largely structured by stochastic processes, when compared to communities of organisms with larger body sizes (i.e., fungi, protists, and nematodes). These differences were partly and significantly explained by differences in organismal dispersal rates. These findings provide insights on how life-history traits relate to the structure of the community of soil organisms, which are often omitted but are directly related to the dynamic interplay of assembly processes. The predictions of how the community of soil organisms varies with body size will deepen our understanding of the ecological mechanism underlying communities in diverse and complex ecosystems.

## Methods

### Site and sampling

A total of 45 soil samples were collected from paddy fields along a north-south transect across East Asia (from September to October in 2010), a rough gradient of latitude from 15.90 to 44.31°N with average annual temperature from 2 to 27.5 °C and average annual precipitation from 550 to 2345 mm. Sampling has been performed as described before^29^, focusing on rice plantations in a transect through East Asia. Twenty soil cores (5 cm diameter and 15 cm depth, free from roots) were randomly collected from 10 × 100 m plots in each site, homogenized in one composite sample and brought to the laboratory on ice. Samples were sieved through a 2-mm mesh and subdivided into two subsamples for determining soil properties and microbial community. Global positioning system (GPS) coordinates recorded at each sampling site were imported into the NOAA website to calculate the mean annual temperature (MAT) and mean annual precipitation (MAP).

### Soil physicochemical characteristics

Soil physicochemical properties were detected according to handbook of soil analysis^47^. Soil pH was determined using a glass electrode in a soil: water ratio of 1:2.5 (w/v). Redox potential was measured by using a combined platinum-calomel electrode connected to a pH/millivolt meter after 10 d waterlogging incubation. Soil organic carbon was titrated against 0.5 M ferrous iron solution after it had been digested with 0.8 M K_2_Cr_2_O_4_ and concentrated H_2_SO_4_ (v/v, 1:1) at 150 °C for 30 min. Total nitrogen was measured as Kjeldahl nitrogen. Briefly, the soil sample was heated and boiled with concentrated H_2_SO_4_. The solution was then absorbed by 2% boric acid solution and titrated against 0.1 M sulfuric acid. Total phosphorus was extracted with HF−HClO_4_ and sodium bicarbonate, and then determined by the molybdenum-blue method. Total potassium was determined using flame emission spectrometry after the soil had been digested in concentrated HF−HClO_4_ (v/v, 2:1). Available phosphorus and available potassium were extracted with sodium bicarbonate and ammonium acetate, respectively, and then determined using the molybdenum-blue method with an atomic absorption spectrophotometer.

### Illumina sequencing and bioinformatic analysis

DNA was extracted from 0.5 g of fresh soil via a Mo-Bio Power Soil DNA Extraction Kit (Mo-Bio Laboratories, Inc., CA, USA) in accordance with the manufacturer’s instructions. DNA quality and quantity were checked using Nano Drop spectrophotometry (Nano Drop Technologies, Wilmington, DE, USA).

The V4−V5 region of bacterial 16S rRNA genes were amplified using the primer pairs 515F (5′-GTGCCAGCMGCCGCGGTAA-3′) and 907R (5′-CCGTCAATTCCTTTGAGTTT-3′)^48^, the internal transcribed spacer (ITS) region of the fungal rRNA gene using the primer pairs ITS1F (5′-CTTGGTCATTTAGAGGAAGTAA-3′) and ITS2 (5′-GCTGCGTTCTTCATCGATGC-3′)^49^, and the 18S rRNA gene of protists using the primer pairs TAReuk454FWD1 (5′-CCAGCASCYGCGGTAATTCC-3′) and TAReukREV3 (5′-ACTTTCGTTCTTGATYRA-3′)^50^. Both the forward and reverse primers were tagged with an adapter and linker sequence, and 8 bp barcode oligonucleotides were added to the forward primer to distinguish the amplicons that originated from different soil samples. Reaction mixtures (20 μl) contained 4 μl of 5× FastPfu Buffer, 0.25 μl of each primer (10 μM), 2 μl of 2.5 mM dNTPs, 10 ng template DNA, and 0.4 μl FastPfu Polymerase. The polymerase chain reaction (PCR) protocol was as follows: an initial pre-denaturation at 95 °C for 5 min; 27 cycles of denaturation at 95 °C for 30 s, annealing at 55 °C for 30 s, and extension at 72 °C for 45 s; and a final extension at 72 °C for 10 min. All amplicons were pooled in equimolar concentrations in a single tube, and then were subjected to library preparation, cluster generation, and sequencing on an Illumina MiSeq platform (Illumina Inc., San Diego, CA).

Raw sequences were quality screened and trimmed using the Quantitative Insights into Microbial Ecology (QIIME) pipeline (v1.9.1)^51^. Sequences that fully matched the barcodes were selected and distributed into separate files. Additional sequence processing was performed, including quality trimming, demultiplexing, and taxonomic assignments. QIIME quality trimming was performed based on the following criteria: (1) sequence reads were truncated before three consecutive low-quality bases were found and re-evaluated for length, and (2) no ambiguous bases were allowed. Chimera detection was performed on assembled reads with UCHIME (v5.1)^52^. The remaining sequences were additionally screened for frame shifts via HMM-FRAME^53^. Finally, sequence reads from each sample were clustered to provide similarity-based operational taxonomic units (OTUs) that had 97% identity cutoffs, and the longest sequence of each OTU was selected as the representative sequence^54^. The taxonomic assignment of the bacterial 16S rRNA sequences was conducted using Silva (release 128)^55^. The fungal ITS sequences were taxonomically assigned using UNITE (version 7)^56^, and the protistan 18S rRNA sequences using PR2^57^. Alpha diversity values and Bray-Curtis dissimilarity for beta-diversity analyses for soil bacterial, fungal and protistan communities were calculated after rarifying all samples to the same sequencing depth.

### Focus taxonomic groups and body size

It is impossible to retrieve body size of each organism owing to unavailable fine-level taxonomic assignation and/or morphological descriptions. A high-throughput morphological detection method to estimate sizes for each organism does not exist. Thus, we distinguished groups based on their taxonomic affiliation at the phylum or subphylum level, and their body sizes were identified through literature estimates based on ~576 dominant genera (Supplementary Data 1). We restricted our analysis to the most abundant phyla (i.e., relative abundance ≥1%) as broadly defined functional traits such as body size and trophic categories are often conserved within phyla, at least being more similar within than across phyla^37,45,46^. Nematodes are grouped by functional traits, because the body size of nematodes is often conserved within functional group^8,58^. Body size of each organism group was identified based on propagule size. In total, body sizes of 576 species were collected from 104 literature studies, which contained 2 studies on 19 bacterial body sizes (cells) of 0.2–5 μm, 53 studies on 271 fungal body sizes (spores) of 1.5–380 μm, 48 studies on 239 protistan body sizes of 3–199 μm, and 1 study on 51 nematode body sizes of 199–1612 μm (Supplementary Data 1).

### Distance-decay slope and halving-distance

To assess the distance-decay of community similarity, we compared log-transformed Bray-Curtis similarity and log-transformed geographic distance matrices. Geographic distances were determined based on the latitude and longitude of each site^59^. The slope of the distance-decay relationship was used to estimate the species turnover rates:

1 $$\log S = a + b\log D$$

where *a* is the intercept and *b* is the slope of the distance-decay relationship.

The halving-distance identifies the distance at which community similarity halves, with a smaller halving distance indicating a faster species turnover^59^. The halving-distance was calculated for each community using a logarithmic decay model:

2 $${{d}}_H = 10^{\frac{{\log \frac{{S_0}}{2} - a}}{b}}$$

where *S*_0_ is the initial community similarity at the lowest transit distance (1 km). Compared with other metrics of dispersal scales, the major advantage of the halving-distance is that it can be calculated for any type of regression between similarity and distance^20,59^. Therefore, it provides a useful and easily comprehensible metric that can be compared across various habitats and organism types.

### Variation partitioning analysis

Variation partitioning analysis was conducted to tease apart the relative importance of environmental factors and spatial factors on variation in microorganism communities. Spatial variables were derived from Moran’s eigenvector maps^60,61^. Briefly, we first conducted a forward selection analysis to select those environmental and spatial variables that were significantly linked with the community composition of different soil organisms. We then performed a variation portioning analysis to calculate the degree of explanation of selected abiotic properties and spatial variables. The explained variance fractions were based on adjusted fractions, which accounted for the number of variables and sample sizes. The significance of each component via partitioning was evaluated with a permutation test, except for the interaction term and residuals.

### Assessment of community assembly processes: β-null deviation

The abundance-based β-null model was used to partition the relative influences of deterministic and stochastic processes mediating community assembly^13,33^. The model evaluated the deviation between the observed β-diversity and the null-expected β-diversity of randomly assembled pair of communities. Based on the calculated species richness and the number of total sequences in each sample, the null scenario was generated by random resampling OTUs and reads in the total community matrix, while preserving the sample richness and number of reads. The total occurrences and abundances of OTU were used as probabilities of selecting an OTU and its associated number of reads, respectively^33,37^. We calculated average Bray-Curtis dissimilarities of 999 simulated communities to obtain null expectations of community dissimilarities for each sample pair. The null deviation value (NDV) is defined as the difference between the observed and averaged null dissimilarities. The NDV value close to 0 indicates higher influence of stochasticity, whereas NDV close to −1 or +1 indicates higher influence of deterministic processes structuring community assembly.

### Assessment of community environmental selection: niche breadth

To evaluate the ecological process of selection, we estimated niche breadth for each organism group with different body sizes. Niche breadth is generally used to evaluate the sensitivity of species to habitat filtering. A species’ niche breadth refers to the suite of environments or resources that it can inhabit or use. Species with a wider niche breadth have stronger metabolic plasticity or higher habitat availability, and are less sensitive to environmental change. Here, we estimated the niche breadth for each OTU by outlying mean index (OMI) analysis. The OMI is a powerful index that determines OTUs’ niche positions and niche breadths by measuring the distance between the mean environmental conditions used by each OTU and the mean environmental conditions of the study area^24,34^. The OMI makes no hypothesis on the shape of species response curves to the environment and gives equal weight to species-poor and species-rich sites. This analysis was conducted with the “niche” function using the “ade4” package in R^24,34^. In brief, we reduced the dimensions of all environmental variables by a principal component analysis. Each microbial OTU was associated with the row profiles of the resulting environmental data, and the average position of each OTU (i.e., niche position) was then calculated along the ordination axes. A low OMI value indicates that a species has a wide niche breadth or high habitat availability, thus suggesting that the species is subjected to lower environmental selection.

### Assessment of community dispersal rate: neutral model

The Sloan neutral model was used to estimate the importance of passive dispersal on community assembly by predicting the relationship between the frequency with which taxa occur in a set of local communities and their abundance across the wider metacommunity. In the model, the dispersal rate (*m*) is a parameter used to evaluate the probability of a random loss of an individual in a local community to be replaced by an immigrant from the metacommunity^62^. It is calculated as follows:

3 $${\mathrm{Freq}}_i = 1 - I\left( {1 \div N{\mathrm{|}}N \times m \times p_i,\;N \times m \times \left( {1 - p_i} \right)} \right)$$

where Freq_*i*_ is the occurrence frequency of taxon *i* across communities; *N* is the number of individuals per community; *m* is the estimated dispersal rate; *p*_*i*_ is the average relative abundance of taxon *i* across communities; and *I()* is the probability density function of beta distribution. This analysis was performed using nonlinear least-squares fitting and the ‘minpack.lm’ package in R^63^. Calculation of 95% CIs for the model predictions was conducted using the Wilson score interval in the ‘Hmisc’ package in R^64^. The overall fit of the model to observed data by comparing the sum of squares of residuals, *SS*_err_, with the total sum of squares, *SS*_total_: model fit = 1–*SS*_err_/*SS*_total_ (generalized *R*-squared)^62^. The fit of the neutral model with the fit of a binomial distribution model was compared to determine whether the model was based on only the random sampling of the source metacommunity. Sampling from a binomial distribution represents the case where local communities are random subsets of the metacommunity in the absence of processes of drift and dispersal limitations. The Akaike information criterion of each model was calculated based on 1000 bootstrap replicates.

### Acquisition of metadata from public databases

We expanded the sampling pool to include more ecosystems to reduce systemic errors, as sampling locations, spatial scale, and methodological quality can affect general relationships between community assembly processes and body size. To best avoid the systemic errors derived from sampling and sequencing and data processing, only six publications with high numbers of samples (a total of 3966 data sets) covering different ecosystems and climatic types were selected to conduct our meta-analysis. Briefly, the meta-analysis data of bacteria come from the Earth Microbiome Project (EMP, http://www.earthmicrobiome.org). Samples processing, sequencing and core amplicon data analysis were performed by the EMP, and all amplicon sequence data and metadata have been made public through the EMP data portal (qiita.microbio.me/emp)^5^. For fungi, the datasets were collected by Bahram et al.^2^ and Davison et al.^65^. For protists, datasets were collected by Fiore-Donno et al.^66^ and Heger et al.^67^. For nematodes, datasets were collected by van den Hoogen et al.^8^. According to the obtained raw abundance value, the β-Null deviation model analysis was carried out, and finally the NDV values of organisms with different body sizes were obtained.

### Statistical analyses

Correlation analyses used in the article were Pearson tests. All statistical analyses were performed in R (R 3.6.2), using “minpack.lm”^63^, “hmisc”^64^, “vegan”^68^, “stats”^69^, and “ade4”^34^ packages.

## Supplementary information

Supplementary Information

Description of Additional Supplementary Information

Supplementary Data

Reporting Summary

## Data Availability

Data used in this work are available from the corresponding authors upon request. The sequences of 16S rRNA gene, 18S rRNA gene, and the ITS region have been deposited in the Sequence Read Archive (SRA) at the National Center for Biotechnology Information (NCBI) with the accession number PRJNA607877, PRJNA608063, and PRJNA608054, respectively. The environmental data and geographical location information of soil samples have been deposited to the figshare database (10.6084/m9.figshare.12622829). Source data are provided with this paper.

## Source data

Source Data

## Code availability

The authors declare that the R (R 3.6.2) codes used to generate the results reported in this study are available in this paper. The R code supporting the findings presented here is available from the GitHub Repository (https://github.com/luluan0522/Code).
