# Supplementary material for: Organism body size structures the soil microbial and nematode community assembly at a continental and global scale
Source: Nat Commun. 2020 Dec 17;11:6406. doi: 10.1038/s41467-020-20271-4 (PMC7747634; doi:10.1038/s41467-020-20271-4)
Supplement: Supplementary file 1 — Supplementary Information [file 41467_2020_20271_MOESM1_ESM.pdf]

Supplementary Information

Organism body size structures the soil microbial and nematode community assembly at a continental and global scale

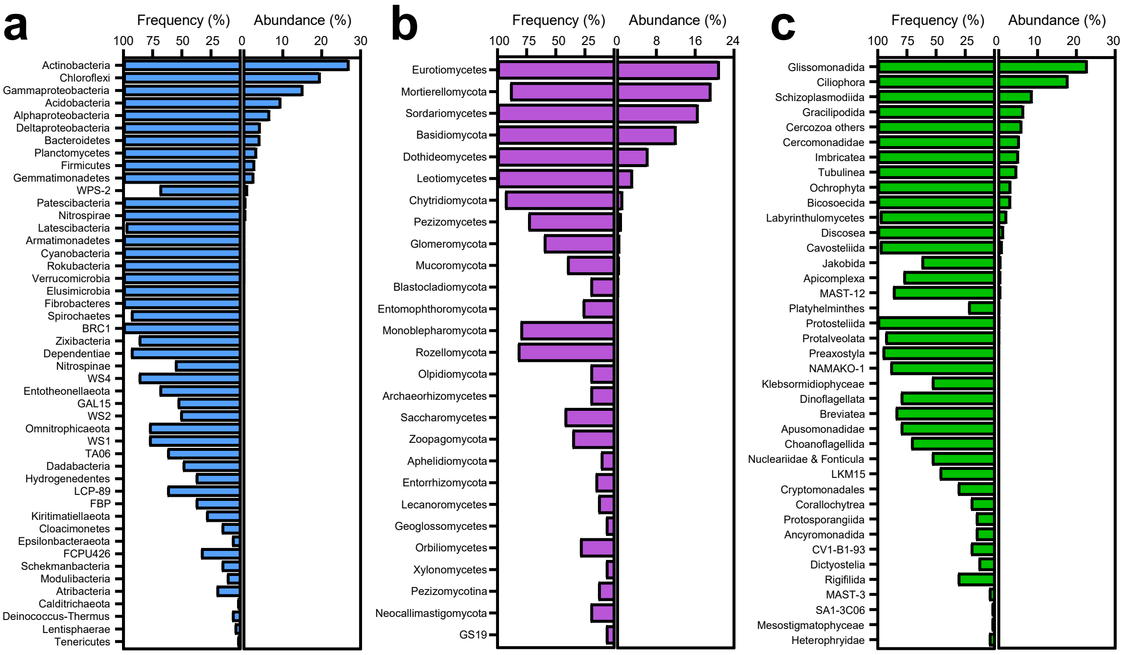

Supplementary Fig. 1. Relative frequency and relative abundance of each group of bacterial (a), fungal (b), and protistan communities (c). Left panel: the proportions of sampling sites at which the groups were present. Right panel: the average relative abundances of each group. Blue, purple and green bars indicate bacteria, fungi and protists, respectively.

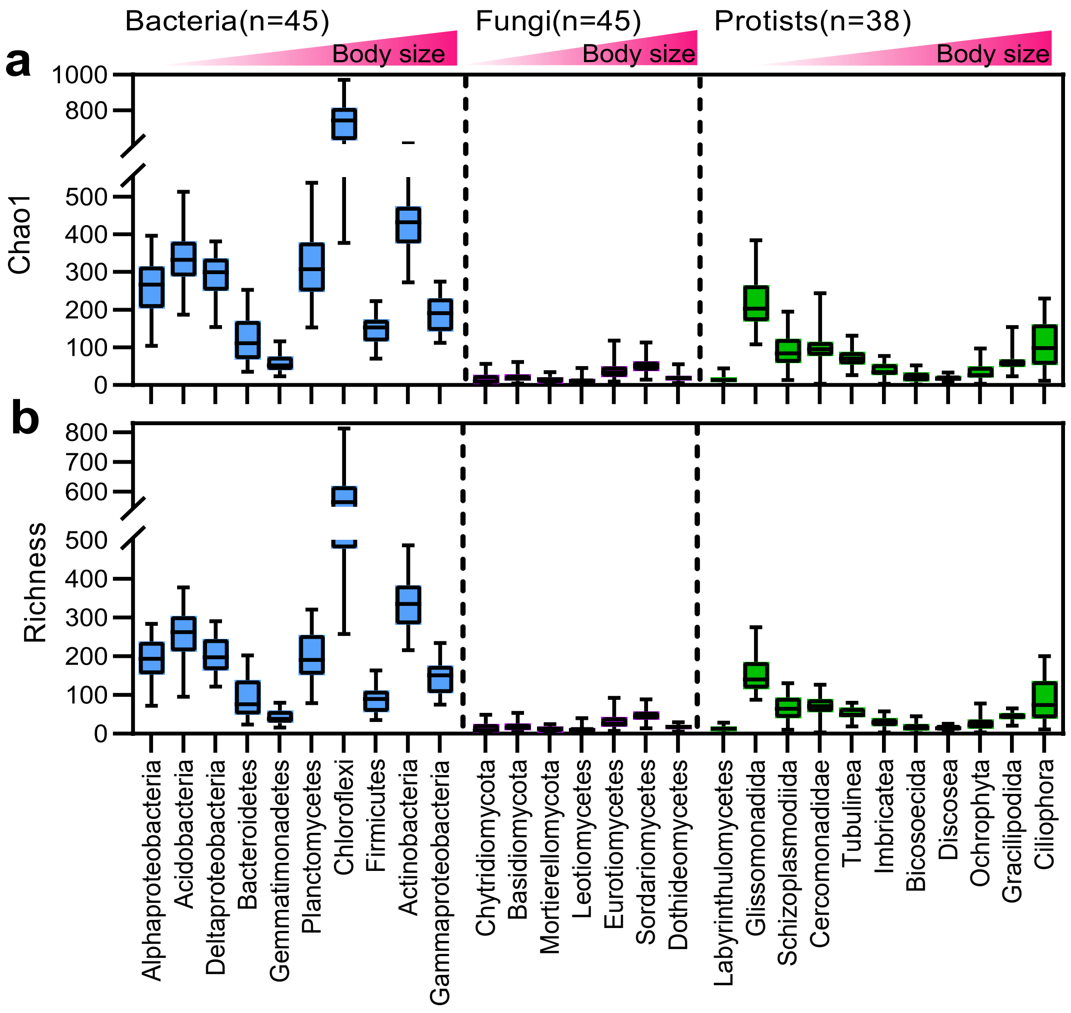

Supplementary Fig. 2. Alpha-diversity indices of soil microbiomes. The Chao1 index (**a**) and richness (**b**) of 28 organism groups, including bacteria (10 groups), fungi (7 groups) and protists (11 groups). The body size of the organism group increases from the left to the right in each plot. Blue, purple and green bars indicate bacteria, fungi and protists, respectively. Box plots indicate median (middle line), 25th, 75th percentile (box) and 5th and 95th percentile (whiskers). n represents the number of samples.

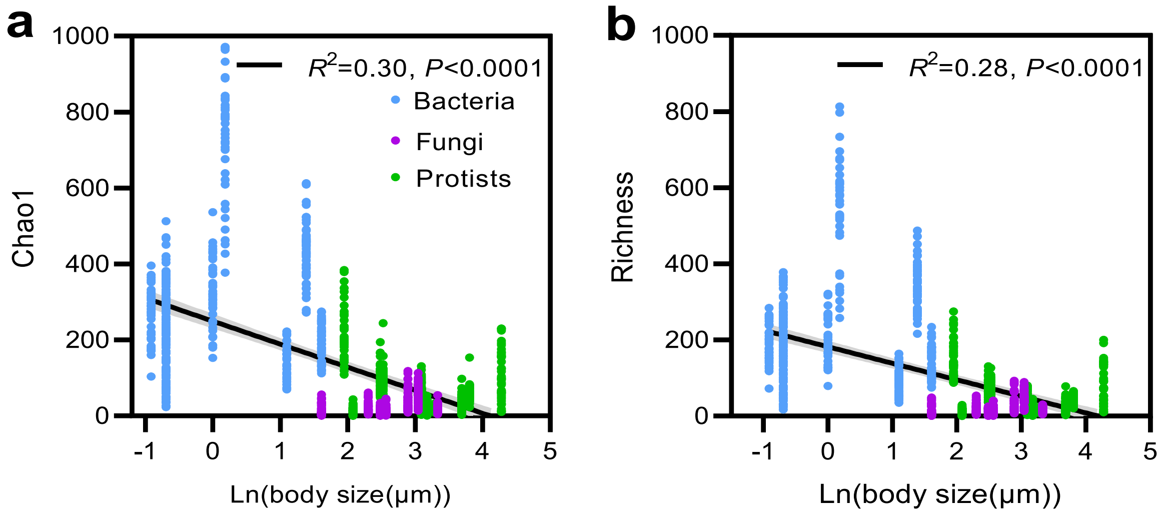

Supplementary Fig. 3. Correlation analyses between alpha-diversity indices and the logarithm of body sizes. The relationships between the logarithm of body sizes and Chao1 index (**a**) and richness (**b**). The statistics are for the regression of all data points. Lines represent the least squares regression fits and shaded areas represent the 95% confidence intervals. The one-sided F test and two-sided t test are applied with the calculated P values.

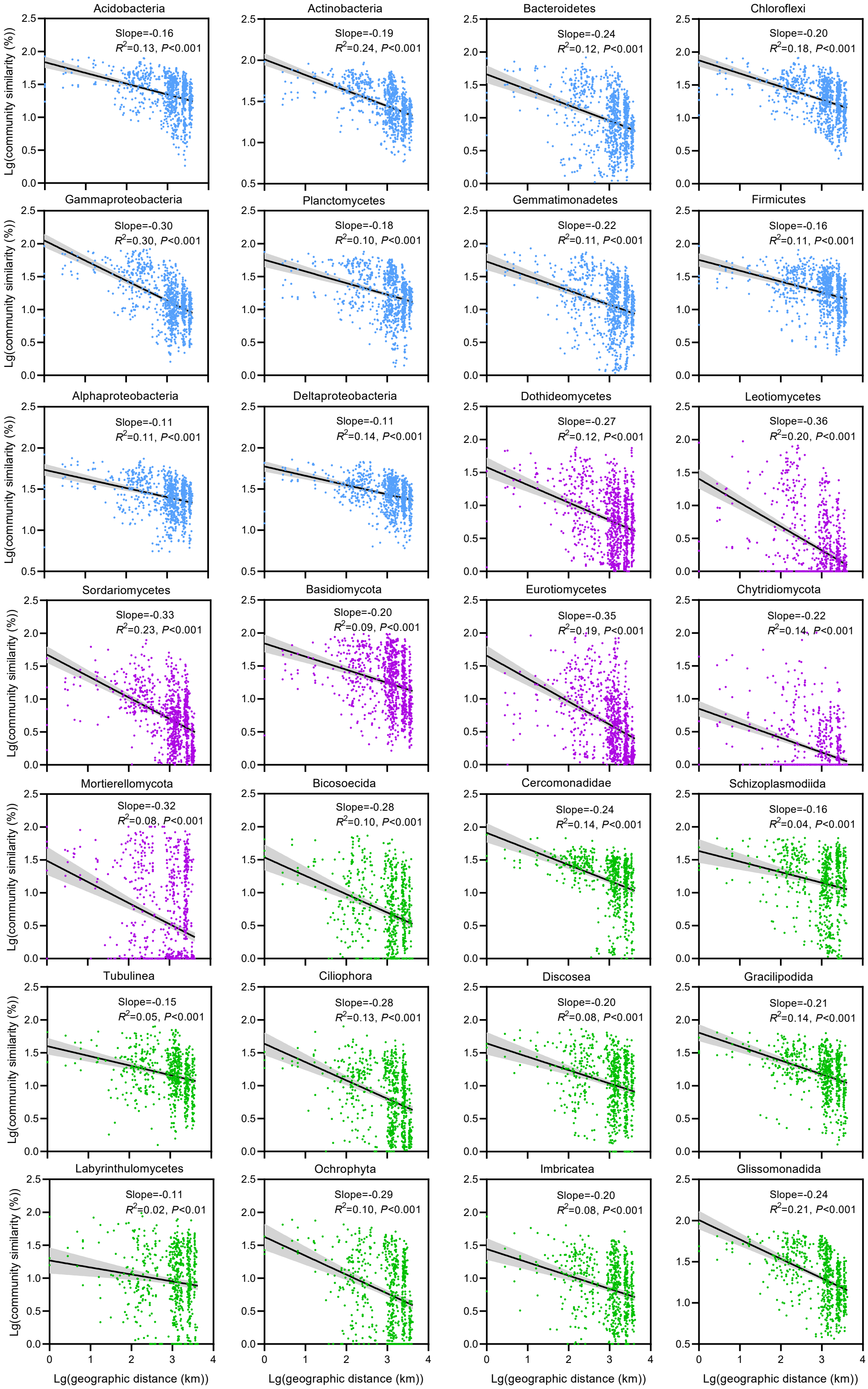

Supplementary Fig. 4. Distance-decay relationships of the 28 organism groups. The 28 organism groups include bacteria (10 groups), fungi (7 groups), and protists (11 groups). Blue,

purple and green indicate bacteria, fungi and protists, respectively. The statistics are for the regression of all data points. Lines represent the least squares regression fits and shaded areas represent the 95% confidence intervals. The one-sided F test and two-sided t test are applied with the calculated P values.

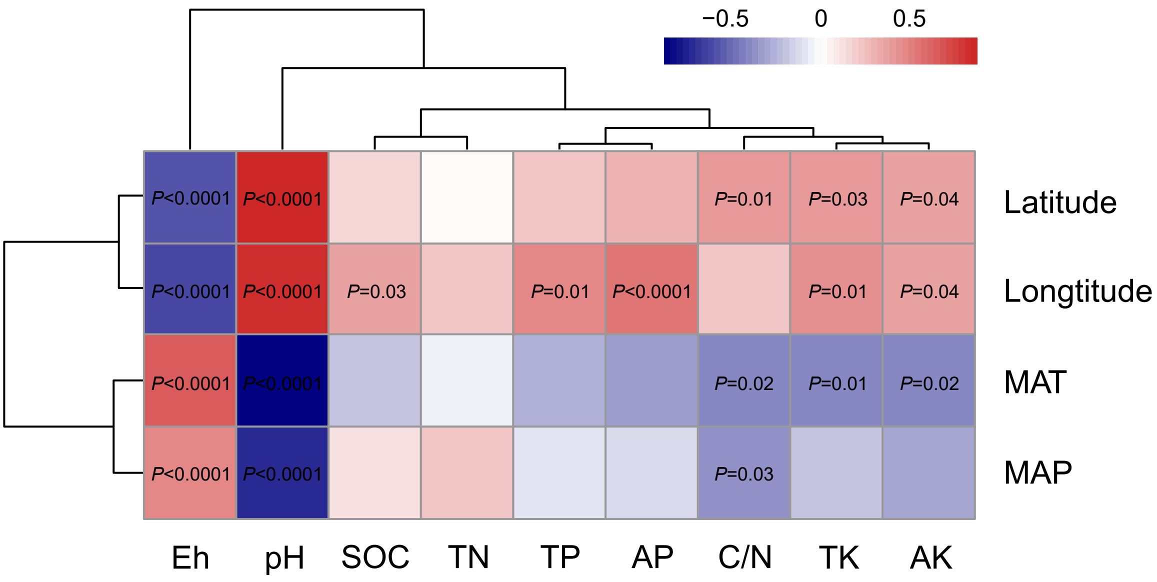

Supplementary Fig. 5. Correlations between soil environmental and spatial variables. Blue colors indicate negative correlation, and red colors indicate positive correlation. The one-sided *F* test and two-sided *t* test are applied with the calculated *P* values.

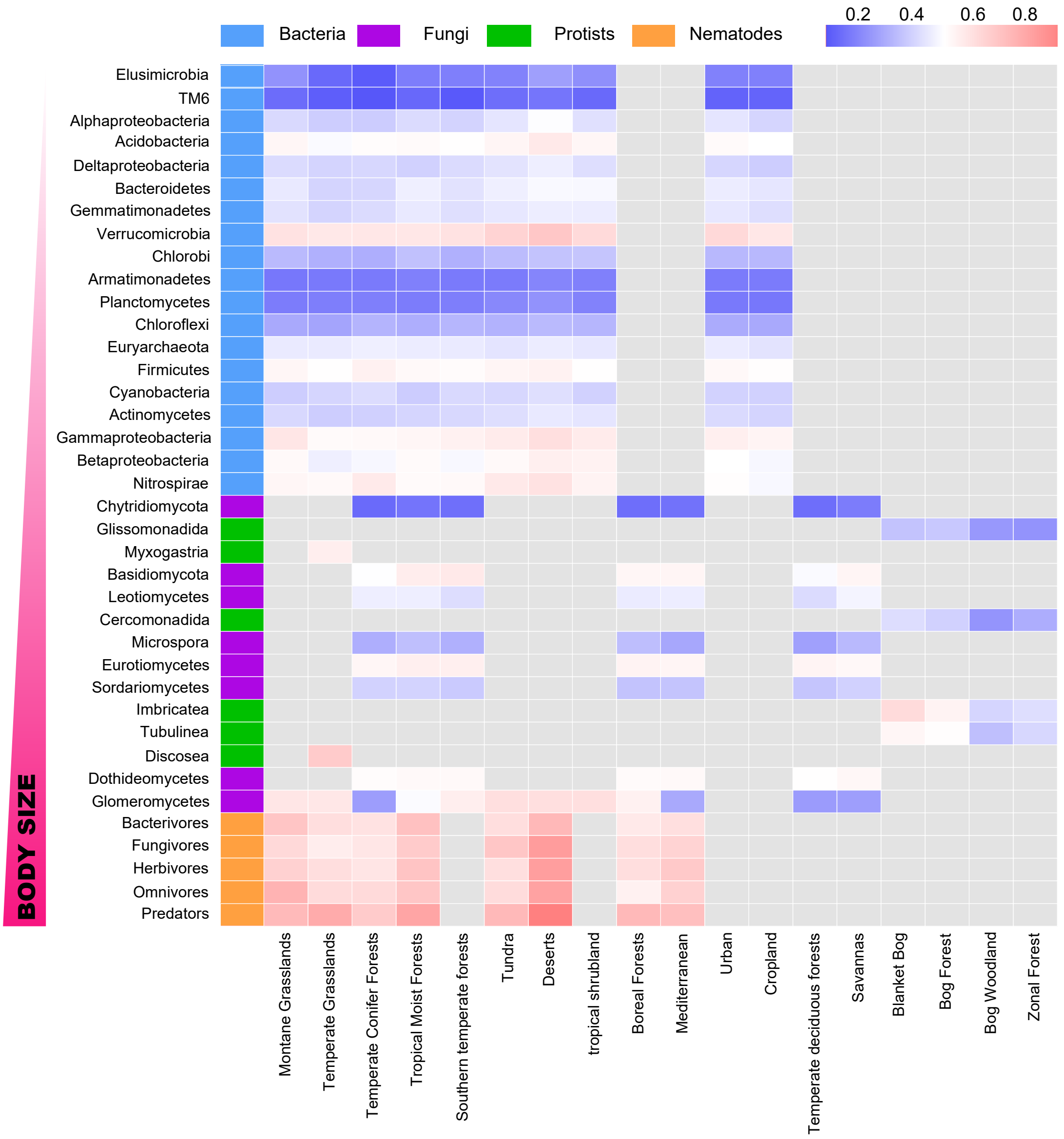

Supplementary Fig. 6. NDVs values of 38 organism groups across distinct ecosystems. The 38 organism groups consist of 19 bacterial groups, 8 fungal groups, 6 protistan groups, and 5 nematode groups. The body size of the organism group increases from the top to the bottom. Grey-shaded cells represent default values.

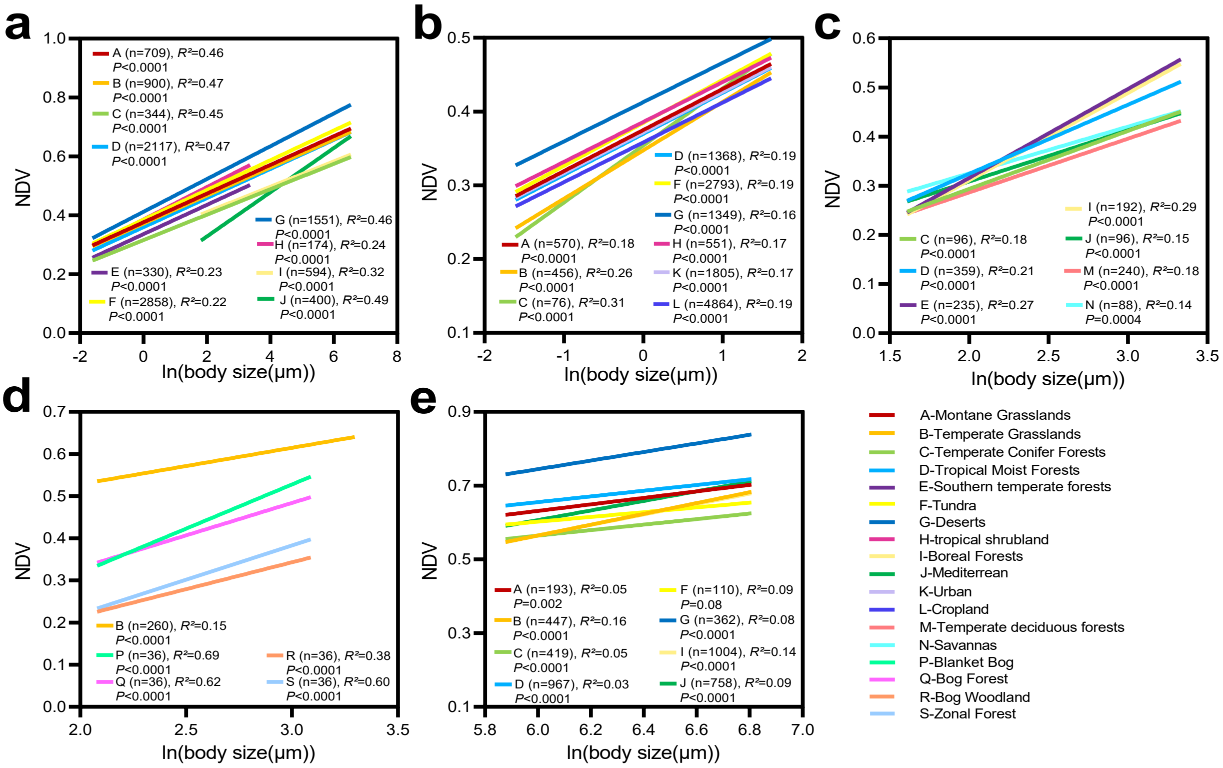

Supplementary Fig. 7. Relationships between NDVs and the logarithm of body sizes. Positive correlations were found when testing all 38 organism groups (**a**), bacterial groups (**b**), fungal groups (**c**), protistan groups (**d**), and nematode groups (**e**) in each ecosystem. Capital letters represent different ecosystems. Each line represents the relationship between the logarithm of body size and NDV. The one-sided *F* test and two-sided *t* test are applied with the calculated *P* values.

Supplementary Table 1. Variation partitioning of community composition according to environmental and spatial variables.

	E (%)		S (%)		E&S	Unexplained	Variable environmental selected
	R^2	P	R^2	P	(%)	(%)	
**Bacteria**							
All	2.55	0.048	9.80	0.001	21.54	66.12	MAP, MAT, pH
Acidobacteria	3.61	0.046	6.16	0.023	19.21	71.03	pH, MAT, TN
Actinobacteria	2.81	0.046	13.09	0.001	25.68	58.42	pH, MAT, MAP
Bacteroidetes	3.74	0.017	9.62	0.001	15.22	71.42	pH, MAT, TP, MAP
Chloroflexi	1.58	0.200	11.62	0.002	19.85	66.95	pH, MAT, MAP
Firmicutes	6.97	0.001	6.97	0.001	11.58	74.49	pH, MAT, TN
Planctomycetes	0.84	0.220	12.05	0.001	15.79	71.31	pH, MAT
Gemmatimonadetes	4.59	0.008	10.51	0.002	15.12	69.78	pH, MAT, AK, TN
Alphaproteobacteria	2.99	0.050	4.40	0.032	16.24	76.37	pH, MAT, AK, TN
Gammaproteobacteria	5.55	0.007	8.87	0.003	24.81	60.77	pH, MAT, MAP, AK, TP
Deltaproteobacteria	1.91	0.080	6.11	0.006	14.96	77.02	pH, MAT
**Fungi**							
All	1.68	0.100	12.78	0.001	10.88	74.66	MAT, MAP, TP
Dothideomycetes	1.83	0.053	14.95	0.001	7.18	76.04	MAT, TN
Leotiomycetes	2.40	0.058	13.73	0.001	7.05	76.82	MAP, MAT, TN, AK
Sordariomycetes	1.17	0.090	13.19	0.001	6.44	79.21	MAT, MAP
Eurotiomycetes	0.67	0.240	12.58	0.001	8.50	78.24	MAT, TP
Basidiomycota	2.88	0.070	11.76	0.003	11.48	73.88	MAT, AP
Chytridiomycota	1.20	0.043	5.81	0.002	3.01	89.97	MAT, TN
Mortierellomycota	2.36	0.150	12.02	0.001	12.62	73.00	MAT, pH, TP, AK, MAP
**Protists**							
All	1.10	0.170	8.85	0.002	10.73	79.30	MAT, MAP
Cercomonadidae	4.90	0.001	2.60	0.032	11.59	80.87	pH, TK, TN, AK
Glissomonadida	2.50	0.250	25.51	0.001	19.92	52.05	MAP, MAT
Imbricatea	3.04	0.032	6.38	0.001	3.67	86.91	MAT, TN, AK
Ciliophora	1.18	0.180	5.71	0.005	8.22	84.88	MAT, TP, MAP
Gracilipodida	5.77	0.010	10.59	0.001	12.59	71.05	MAT, MAP, TN, pH
Tubulinea	2.41	0.037	8.83	0.001	8.16	80.60	MAT, TP
Schizoplasmodiida	2.43	0.049	3.85	0.046	2.90	90.82	AK, AP
Bicosoecida	1.65	0.110	6.95	0.005	6.95	84.45	MAT, AP
Discosea	4.31	0.001	19.47	0.001	7.25	68.97	MAT, MAP
Labyrinthulomycetes	7.25	0.001	4.63	0.003	3.28	84.84	AP, pH
Ochrophyta	1.07	0.590	9.60	0.001	13.48	75.85	MAT, MAP, TN, TP

Values correspond to the fractions explained (R^2) by pure environmental (E), and spatial (S) effects, as well as combined effect (E&S). Significance of pure effects E, S and of the full model was obtained with 1000 Monte Carlo permutations. Intersection effects are not testable. Variables retained after the forward selection procedure are shown by order of contribution as determined by pRDA. The significance of each component via partitioning was evaluated with a permutation test.

Supplementary Table 2. Parameters and fits of null models across the 28 organism groups.

	m	m.ci	m.mle	Rsqr	RMSE	AIC	BIC
Bacteria							
All	0.089	0.003	0.090	0.49	0.15	22363	22378
Actinobacteria	0.081	0.008	0.081	0.58	0.18	-634	-624
Chloroflexi	0.068	0.004	0.068	0.48	0.14	-2930	-2918
Gammaproteobacteria	0.042	0.006	0.042	0.46	0.17	-470	-461
Acidobacteria	0.084	0.009	0.084	0.47	0.17	-600	-590
Alphaproteobacteria	0.092	0.011	0.092	0.55	0.16	-624	-615
Deltaproteobacteria	0.211	0.022	0.211	0.65	0.14	-845	-836
Bacteroidetes	0.043	0.007	0.043	0.46	0.15	-527	-518
Planctomycetes	0.251	0.032	0.251	0.44	0.13	-1389	-1379
Firmicutes	0.271	0.065	0.271	0.31	0.18	-206	-199
Gemmatimonadetes	0.024	0.005	0.024	0.45	0.13	-309	-302
Fungi							
All	0.005	0.000	0.001	0.05	0.10	8308	8321
Eurotiomycetes	0.004	0.001	0.004	0.00	0.14	2618	2625
Mortierellomycota	0.002	0.000	0.000	0.04	0.18	2532	3540
Sordariomycetes	0.001	0.000	0.001	0.12	0.12	964	972
Basidiomycota	0.000	0.000	0.000	0.25	0.08	3616	3623
Dothideomycetes	0.002	0.001	0.002	0.22	0.14	-151	-145
Leotiomycetes	0.001	0.000	0.001	0.00	0.08	543	549
Chytridiomycota	0.001	0.000	0.001	0.01	0.06	3791	3799
Protists							
All	0.023	0.002	0.776	0.14	0.16	10155	10168
Cercozoa	0.043	0.004	0.747	0.32	0.16	3781	3792
Ciliophora	0.011	0.003	0.853	0.02	0.18	940	948
Schizoplasmodiida	0.030	0.006	0.251	0.35	0.16	3973	3981
Gracilipodida	0.012	0.003	0.853	0.13	0.18	412	419
Tubulinea	0.053	0.008	0.774	0.57	0.11	4132	4140
Ochrophyta	0.008	0.003	0.836	0.03	0.15	375	381
Bicosoecida	0.012	0.004	0.797	0.07	0.13	256	261
Labyrinthulomycetes	0.004	0.001	0.846	0.04	0.14	225	231
Discosea	0.021	0.009	0.691	0.15	0.15	205	210
Cercomonadidae	0.055	0.010	0.701	0.47	0.15	569	576
Glissomonadida	0.078	0.010	0.703	0.46	0.15	1596	1605
Imbricatea	0.010	0.003	0.047	0.08	0.17	367	373

m is a single free parameter describing the migration rate, which is interpreted as the influence of drift and dispersal; m.ci and m.mle are the fitting parameters of *m*; Rsqr, Root Mean Square Error (RMSE), Akaike information criterion (AIC), and Bayesian Information Criterion (BIC) are used to assess the overall fit of the model.
