# Supplementary material for: Organism body size structures the soil microbial and nematode community assembly at a continental and global scale
Source: Nat Commun. 2020 Dec 17;11:6406. doi: 10.1038/s41467-020-20271-4 (PMC7747634; doi:10.1038/s41467-020-20271-4)
Supplement: Supplementary file 2 — Description of Additional Supplementary Information [file 41467_2020_20271_MOESM2_ESM.pdf]

Description of Additional Supplementary Files

File Name: Supplementary Data 1

Description: Summary table of body sizes.
