# Supplementary material for: Organism body size structures the soil microbial and nematode community assembly at a continental and global scale
Source: Nat Commun. 2020 Dec 17;11:6406. doi: 10.1038/s41467-020-20271-4 (PMC7747634; doi:10.1038/s41467-020-20271-4)
Supplement: Supplementary file 4 — Reporting Summary [file 41467_2020_20271_MOESM4_ESM.pdf]

nature research

Corresponding author(s): Prof. Yuji Jiang

Last updated by author(s): Nov 16, 2020

Reporting Summary

Nature Research wishes to improve the reproducibility of the work that we publish. This form provides structure for consistency and transparency in reporting. For further information on Nature Research policies, see Authors & Referees and the Editorial Policy Checklist.

Statistics

For all statistical analyses, confirm that the following items are present in the figure legend, table legend, main text, or Methods section.

n/a	Confirmed	
☐	☒	The exact sample size (*n*) for each experimental group/condition, given as a discrete number and unit of measurement
☐	☒	A statement on whether measurements were taken from distinct samples or whether the same sample was measured repeatedly
☐	☒	The statistical test(s) used AND whether they are one- or two-sided<br>*Only common tests should be described solely by name; describe more complex techniques in the Methods section.*
☐	☒	A description of all covariates tested
☐	☒	A description of any assumptions or corrections, such as tests of normality and adjustment for multiple comparisons
☐	☒	A full description of the statistical parameters including central tendency (e.g. means) or other basic estimates (e.g. regression coefficient) AND variation (e.g. standard deviation) or associated estimates of uncertainty (e.g. confidence intervals)
☐	☒	For null hypothesis testing, the test statistic (e.g. *F*, *t*, *r*) with confidence intervals, effect sizes, degrees of freedom and *P* value noted<br>*Give P values as exact values whenever suitable.*
☒	☐	For Bayesian analysis, information on the choice of priors and Markov chain Monte Carlo settings
☒	☐	For hierarchical and complex designs, identification of the appropriate level for tests and full reporting of outcomes
☐	☒	Estimates of effect sizes (e.g. Cohen's *d*, Pearson's *r*), indicating how they were calculated

Our web collection on statistics for biologists contains articles on many of the points above.

Software and code

Policy information about availability of computer code

Data collection	We conducted continental sampling (45 samples) and performed a global literature meta-analysis (3966 datasets). The body sizes of organism groups were obtained from the published references (including 576 species) as shown in “supplementary Data” file. No software was used for data collection.
Data analysis	For Bioinformatic analysis, the software Qiime (v1.9.1) and UCHIME (v5.1) and HMM-FRAME were used. All statistical analyses were performed in R (v3.5.1; http://www.r-project.org/), using minpack.lm, hmisc, vegan, stats, and ade4 packages. The R code supporting the findings presented here is available from the GitHub Repository (https://github.com/luluan0522/Code).

For manuscripts utilizing custom algorithms or software that are central to the research but not yet described in published literature, software must be made available to editors/reviewers. We strongly encourage code deposition in a community repository (e.g. GitHub). See the Nature Research guidelines for submitting code & software for further information.

Data

Policy information about availability of data

All manuscripts must include a data availability statement. This statement should provide the following information, where applicable:

- Accession codes, unique identifiers, or web links for publicly available datasets
- A list of figures that have associated raw data
- A description of any restrictions on data availability

Data used in this work are available from the corresponding authors upon request. The sequences of 16S rRNA gene, 18S rRNA gene, and the ITS region have been deposited in the Sequence Read Archive (SRA) at the National Center for Biotechnology Information (NCBI) with the accession number PRJNA607877 (https://www.ncbi.nlm.nih.gov/search/all/?term=PRJNA607877), PRJNA608063 (https://www.ncbi.nlm.nih.gov/search/all/?term=PRJNA608063), and PRJNA608054 (https://www.ncbi.nlm.nih.gov/search/all/?term=PRJNA608054), respectively. The environmental data and geographical location information of soil samples have been deposited to the figshare database (http://doi.org/ 10.6084/m9.figshare.12622829). The source data underlying Figs. 2, 3a–e, 4a–j, and 5a–e, and Supplementary Figs. 1a–c, 2a–b, 3a–d, 4, 5, 6, and 7a–e are provided as a Source Data file.

Field-specific reporting

Please select the one below that is the best fit for your research. If you are not sure, read the appropriate sections before making your selection.

☐ Life sciences ☐ Behavioural & social sciences ☒ Ecological, evolutionary & environmental sciences

For a reference copy of the document with all sections, see nature.com/documents/nr-reporting-summary-flat.pdf

Ecological, evolutionary & environmental sciences study design

All studies must disclose on these points even when the disclosure is negative.

Study description	We performed a continental sampling followed by a global literature meta-analysis to provide the first holistic cross-microbiome study that links biogeography with microbial traits, here body size.
Research sample	In this study, we intended to investigate the extent to which distinct ecological processes mediate the community assembly of differentially sized microbial organism groups. The continental scale environmental sampling approach (spanning 4,165 km) including 45 surface soil samples (0-15 cm) and a global-scale meta-analysis (3,966 datasets) are sufficient to compare the community assembly of differentially sized microbes and nematodes. All the rice planting continued for 30-50 years (even for more than hundred years). The soil sampling sites with a rice planting area more than 5 ha had consistent tillage and irrigation practices, and similar soil texture and terrain without crop patchiness. Specifically, a total of 45 surface soil samples (0-15 cm) were collected from paddy fields to provide the holistic cross-microbiome study that links biogeography with microbial traits, i.e. body size here. Soil bacterial, fungal, and protistan community assembly was determined by sequencing of 16S rRNA, ITS, and 18S rRNA amplicons. The community assembly of 38 organism groups was compared by available published sequence data (3,966 datasets) including bacterial, fungal, protistan, and nematode communities. The body sizes of all 45 organism groups were calculated by available published body size (576 species) including bacteria, fungi, protists and nematodes.
Sampling strategy	During the sampling period, the water layer was drained but soil water content was near field capacity. Twenty soil cores (5 cm diameter and 15 cm depth, free from roots) were randomly collected from 10 × 100 m plots in each site, homogenized in one composite sample and brought to the laboratory on ice. Samples were sieved through a 2 mm mesh and subdivided into two subsamples for determining soil properties and microbial community. In total, we have collected enough samples and replicates for the statistical analyses of soil chemical properties and microbiome assembly.
Data collection	All sample collection was performed by authors YJ, YS and BS. Soil chemical analysis was carried out by YJ, LL, MC following standard protocols. Soil DNA preparation and MiSeq sequencing analysis were carried out by YJ, LL, and MC. Raw sequence data for meta-analysis were downloaded from NCBI SRA using SRA toolkit prefetch, and raw sequences were then converted into fastq format using SRA toolkit fastq-dump for downstream analyses.
Timing and spatial scale	A total of 45 soil samples were collected from paddy fields along a north-south transect across East Asia (from September to October in 2010), a rough gradient of latitude from 15.90 to 44.31°N.
Data exclusions	No data was excluded from the analyses.
Reproducibility	All data supporting our conclusions and codes dealing with these data have been deposited into public databases for open and free use, which can well guarantee the reproducibility of our findings.
Randomization	At each sampling site, we established transects in a 10 m × 100 m rectangular plot. Three sub-plots were randomly placed at least 40 m apart along the transect, and each sub-plot was in a circle with a 5 m diameter. Within each sub-plot, 20 soil cores (5 cm diameter) of the upper 15 cm soils were collected randomly and composited into a single bulk sample.
Blinding	Since there are no experimental groups in our analyses, blinding was not relevant to this study.

Did the study involve field work? ☒ Yes ☐ No

Field work, collection and transport

Field conditions	Soil samples were collected from paddy fields, along a north-south transect across East Asia, with average annual temperature from 2 to 27.5 ℃ and average annual precipitation from 550 to 2345 mm. During the sampling period, the water layer was drained but soil water content was near field capacity.
Location	Sampling area along a north-south transect across East Asia, a rough gradient of latitude from 15.90 to 44.31°N.
Access and import/export	Samples were collected by all authors in their respective locations, which obtains local permits from Institute of Soil Science, Chinese Academy of Sciences at September 2010.
Disturbance	This study did not cause any environmental disturbance.

Reporting for specific materials, systems and methods

We require information from authors about some types of materials, experimental systems and methods used in many studies. Here, indicate whether each material, system or method listed is relevant to your study. If you are not sure if a list item applies to your research, read the appropriate section before selecting a response.

Materials & experimental systems

n/a	Involved in the study
☒	☐ Antibodies
☒	☐ Eukaryotic cell lines
☒	☐ Palaeontology
☒	☐ Animals and other organisms
☒	☐ Human research participants
☒	☐ Clinical data

Methods

n/a	Involved in the study
☒	☐ ChIP-seq
☒	☐ Flow cytometry
☒	☐ MRI-based neuroimaging
